# Validation of Patient-Specific Cerebral Blood Flow Simulation Using Transcranial Doppler Measurements

**DOI:** 10.3389/fphys.2018.00721

**Published:** 2018-06-19

**Authors:** Derek Groen, Robin A. Richardson, Rachel Coy, Ulf D. Schiller, Hoskote Chandrashekar, Fergus Robertson, Peter V. Coveney

**Affiliations:** ^1^Department of Computer Science, Brunel University London, London, United Kingdom; ^2^Centre for Computational Science, University College London, London, United Kingdom; ^3^Centre for Mathematics and Physics in the Life Sciences and Experimental Biology, University College London, London, United Kingdom; ^4^Department of Materials Science and Engineering, Clemson University, Clemson, SC, United States; ^5^School of Health Research, Clemson University, Clemson, SC, United States; ^6^Lysholm Department of Neuroradiology, National Hospital for Neurology and Neurosurgery, University College London, London, United Kingdom

**Keywords:** lattice-Boltzmann, middle cerebral artery, computational fluid dynamics, transcranial Doppler, high performance computing, blood flow, validation study

## Abstract

We present a validation study comparing results from a patient-specific lattice-Boltzmann simulation to transcranial Doppler (TCD) velocity measurements in four different planes of the middle cerebral artery (MCA). As part of the study, we compared simulations using a Newtonian and a Carreau-Yasuda rheology model. We also investigated the viability of using downscaled velocities to reduce the required resolution. Simulations with unscaled velocities predict the maximum flow velocity with an error of less than 9%, independent of the rheology model chosen. The accuracy of the simulation predictions worsens considerably when simulations are run at reduced velocity, as is for example the case when inflow velocities from healthy individuals are used on a vascular model of a stroke patient. Our results demonstrate the importance of using directly measured and patient-specific inflow velocities when simulating blood flow in MCAs. We conclude that localized TCD measurements together with predictive simulations can be used to obtain flow estimates with high fidelity over a larger region, and reduce the need for more invasive flow measurement procedures.

## 1. Introduction

Computational fluid dynamics (CFD) has been widely applied by researchers to model blood flow in cerebral arteries and specifically within aneurysms (Cebral et al., 2011; Miura et al., 2013; Mountrakis et al., 2013; Byrne et al., 2014; Ouared et al., 2016). There is considerable interest in exploring the correlation between the dynamical properties of blood flow and clinical outcomes, with the long-term aim to provide a personalized, predictive simulation approach for aneurysm formation, growth, and/or rupture (Jou et al., 2008; Bernabeu et al., 2013; Xiang et al., 2014). When performing such simulations it is essential that computational models are able to deliver a realistic prediction of patient-specific flow velocities.

A range of simulation studies have been performed using patient-specific flow measurements derived from phase contrast magnetic resonance angiography (pc-MRA, see e.g., Boussel et al., 2008). However, Marzo et al. (2011) found that using this type of measurement provides limited accuracy benefits in comparison with modeled boundary conditions. The use of CFD in combination with transcranial Doppler (TCD) velocity measurements has been less extensively researched (see e.g., Hassan et al., 2004), primarily because reliable TCD measurements can only be made in a limited subset of the cerebral arteries. In addition, TCD measurements with hand-held devices may contain errors if held at an incorrect angle (e.g., an underprediction of approximately 1.6% if the angle is off by 10 degrees). However, the excellent time resolution of TCD allows for a more reliable detection of peak velocities, and helps to establish more precise pulsatile flow profiles. Indeed, the maximum velocity values found by TCD are frequently around 30% higher than those found through pc-MRA (Chang et al., 2011; Meckel et al., 2013). In addition, TCD is non-invasive, unlike pc-MRA, and both are widely applied in clinical settings today.

Blood consists of blood cells which reside within a liquid medium known as blood plasma. Blood has a viscosity which decreases under shear flow (shear-thinning), unlike water which exhibits a constant Newtonian viscosity regardless of shear strain rate. Many well-known CFD studies of cerebrovascular blood flow are performed using a Newtonian fluid model (e.g., Cebral et al., 2011; Miura et al., 2013; Byrne et al., 2014), though recent research has found that such an assumption could lead to over-estimation of wall shear stresses (WSS) in cerebral arteries and aneurysms (Bernsdorf and Wang, 2009; Xiang et al., 2011; Khan et al., 2016). As a result, it can also alter the outcome of related diagnostic techniques such as three-band diagram analysis (Bernabeu et al., 2013), a technique proposed by Chatzizisis et al. (2008) to compare WSS at a specific location, over a period of time, to a set of pathological threshold values.

Existing CFD studies of cerebrovascular flow frequently derive inflow velocities not from the specific patient of interest, but from healthy subjects (e.g., Miura et al., 2013; Byrne et al., 2014) or idealized pulsatile profiles (Womersley flow, e.g., Castro et al., 2006; Alnæs et al., 2007; Cebral et al., 2011). However, blood flow velocities in middle cerebral arteries (MCA) from healthy subjects are typically much lower than those from stroke patients or patients suffering from hypertension. In this context Venugopal et al. (2007) found that mean WSS properties of simulations at Reynolds numbers (Re) below 200 do not correspond in any linear way to WSS properties of simulations at Re = 340–675. Itani et al. (2015) investigated how the mean, maximum, and minimum wall shear stress changes when a patient is subject to different levels of exercise intensity. They also found a non-linear relation between maximum inflow velocity and extracted WSS.

In this work, we simulate blood flow in a patient-specific MCA model using patient-specific TCD measurements as inflow boundary conditions, and compare our predictions against clinical measurements at four locations. Our simulations employ the lattice-Boltzmann method at high resolution, a technique which has been shown by Jain et al. among others, to be particularly well-suited for simulating cerebrovascular and aneurysmal blood flow Jain et al. (2016). We perform simulations imposing the measured velocity from the individual patients at the inlet, and investigate how the choice of rheology model affects the predicted flow velocities throughout the MCA. In addition, we report on the accuracy of velocity predictions when running simulations with downscaled inlet velocities, and rescaling the velocities obtained from the measurement planes.

## 2. Materials and methods

**Figure 1:**
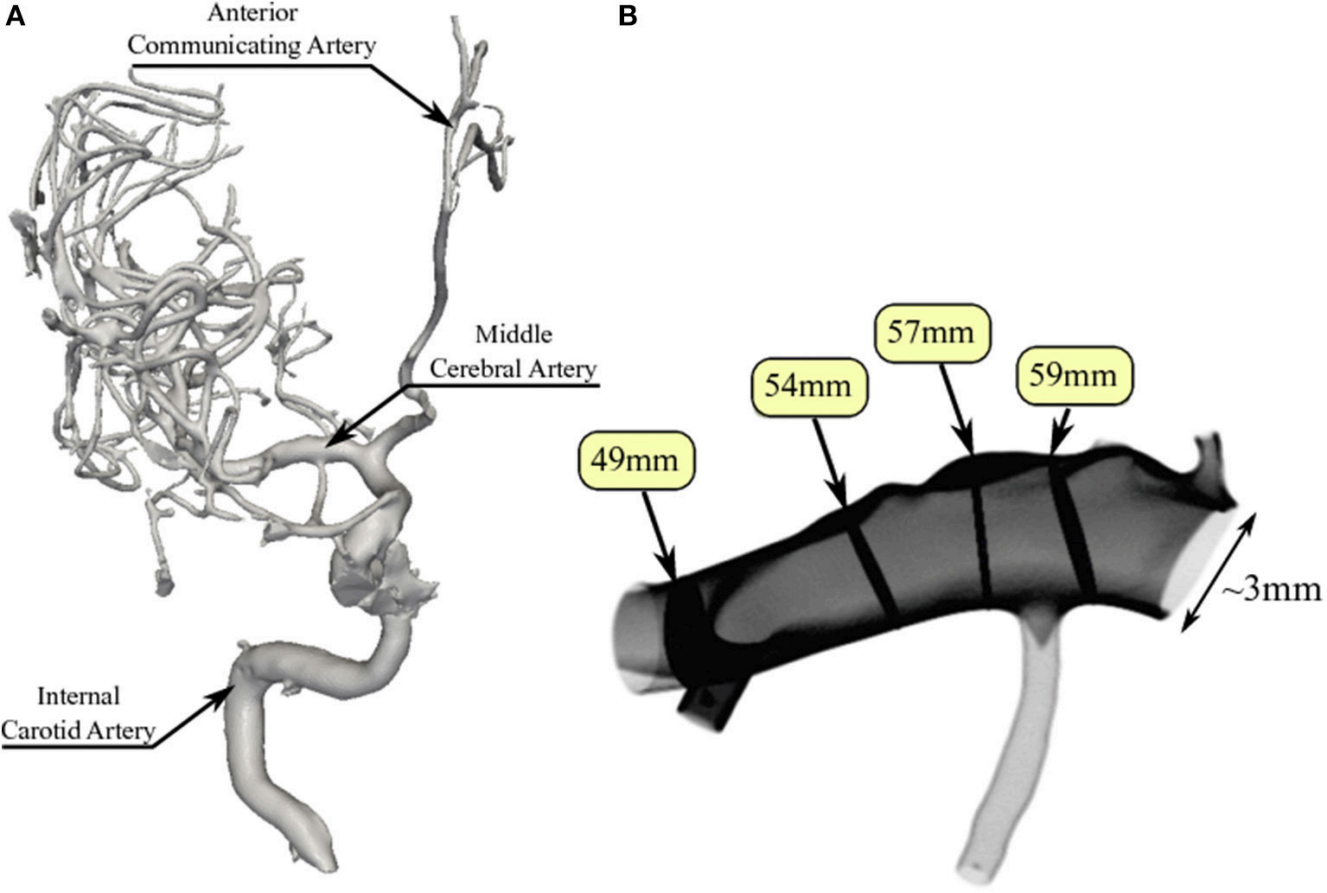
Overview of the overall patient vasculature in the medical images **(A)**, and the patient-specific 3D model used in our simulations **(B)**. As part of the simulation model overview, we indicate the four TCD measurement planes used for validation. Both the inlet and the 63 mm TCD measurement plane are at the right hand side of the image.

**Figure 2:**
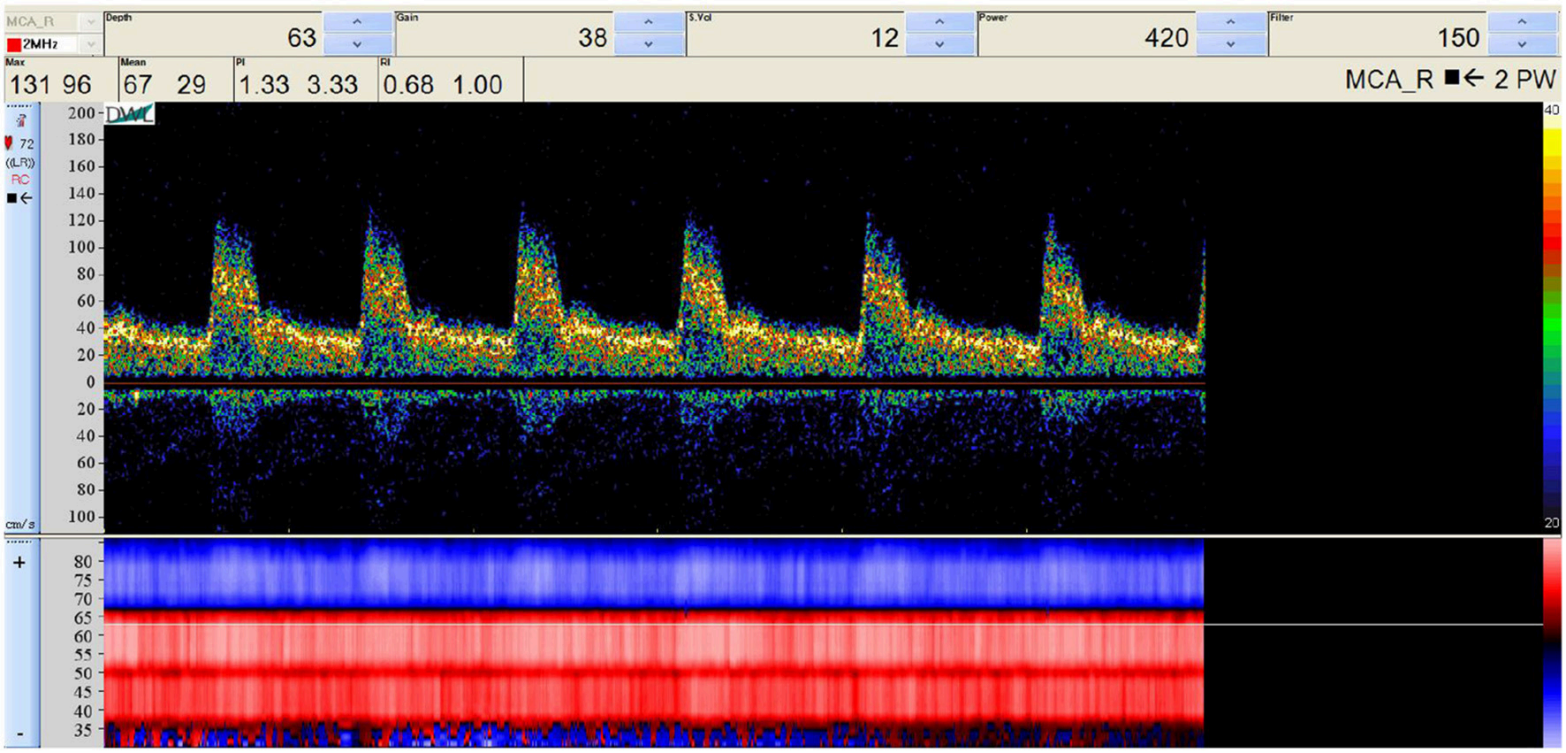
Raw TCD input image of the measured velocity at a depth of 63 mm (inflow boundary plane). The measured velocity at the selected depth (63 mm) is given at the top, while the general flow direction at all depths is given at the bottom, either toward the device (red) or away from it (blue). We observe a change in flow direction around a depth of 67 mm, which is at the junction between the right MCA and the right ACA.

To perform our simulations, we use the HemeLB software (Groen et al., 2013; Nash et al., 2014) for lattice Boltzmann simulations of blood flow in cerebral arteries. The lattice Boltzmann method (LBM) is a highly scalable simulation approach which uses a discretized kinetic model on a regular lattice to reproduce the dynamics of incompressible fluid flow. The LBM can be interpreted as a numerical solver for the Navier-Stokes equation with the advantage that it algorithmically separates the non-linearity from the non-locality. Specific *boundary conditions* are applied to create accurate representations of fluid flow near vessel walls, as well as inflow and outflow boundaries. In our case, we adopt a 3-dimensional LBM which propagates fluid flow in 19 directions per grid point (D3Q19) using a BGK collision operator (see e.g., Succi, 2001 for details). For the boundary conditions, we used the Bouzidi (Bouzidi et al., 2001) model to represent flow interactions with the vessel walls. Patient-specific inflow conditions were obtained from TCD measurements performed at the National Hospital for Neurology and Neurosurgery (NHNN) using the Doppler BoxX (with a handheld device) from the DWL company, and used rotational angiography data from NHNN to obtain imaging data from the same patient. TCD measurements were recorded for at least six cardiac cycles each in the right MCA, consecutively at depths of 49, 54, 57, 59, and 63 mm away from the temple area (see Figure 1 for the location of the TCD validation planes in the 3D model, Table 1 for the velocity measurements, and Figure 2 for the TCD image measurement at the inflow boundary). The Doppler BoxX provides a flow direction indication at all depths whenever a measurement is made. In our case, this feature enabled us to hold the TCD device such that the flow was observable in the right MCA, as well as the right Anterior Communicating Artery (ACA). This is important, because retaining such a tight orientation minimizes TCD measurement errors caused by holding the device at a wrong angle. In addition, to align the TCD measurements precisely with the corresponding planes of flow direction in the simulation domain, we performed a triangulation and an angle correction with respect to the perpendicular flow direction (see Table 2 for our triangulation results). The maximum velocity at the inflow boundary, extracted from the TCD data, was 1.50 m/s.

**Table 1:** Overview of measured and simulated flow characteristics in the MCA, as well as relative differences between measurement and simulation.

**Depth [mm]**	**49**	**54**	**57**	**59**	**63 (inflow)**
Mean cycle length [s]	0.930	0.786	0.906	0.804	0.894
Maximum cycle length [s]	0.972	0.822	0.972	0.822	0.978
Minimum cycle length [s]	0.870	0.708	0.828	0.786	0.816
$v_{\text{max}}^{\text{TCD}}$ [m/s]	1.43	1.61	1.32	1.26	1.50
$v_{\text{max}}^{\text{sim,Newton}}$ [m/s]	1.32	1.50	1.27	1.37	1.50*
$d_{\text{r}}^{\text{Newton}}$	−7.7%	−6.8%	−3.8%	+8.7%	−
$v_{\text{max}}^{\text{sim,CY}}$ [m/s]	1.32	1.51	1.27	1.37	1.50*
$d_{\text{r}}^{\text{CY}}$	−7.7%	−6.2%	−3.8%	+8.7%	−

In rows 1, 2, and 3 we report the mean, maximum and minimum cardiac cycle length extracted from the TCD velocity measurements, respectively. In row 4 we provide the maximum velocity (v_max_) as measured in the TCD data, and in rows 5 and 7 we present v_max_ for our (full velocity) HemeLB simulations with the Newtonian and the CY rheology models, respectively. Relative differences (d_r_) between the TCD measurements and each of the respective two HemeLB simulations are in rows 6 and 8. We use the velocity obtained from TCD as the inflow condition for our simulation. *Indicates simulation values which are preset (boundary conditions).

**Table 2:** Triangulation points, input, output, and measurement plane locations (and orientations where applicable) in the simulation domain, used to calculate the angle correction.

	**Distance from TCD device (mm)**	**Location (4 d.p.)**	**Normal (4 d.p.)**
Triangulation point 1	45	[35.5,−203.8,−154.658]	−
Triangulation point 2	50	[22.3,−209.8,−156 ]	−
Triangulation point 3	66	[35.3,−203.8,−154.658]	−
Measurement plane 1	49	[21,−210.2,−156.5]	[0.9474,0.2650,0.1796]
Measurement plane 2	54	[25.4,−207.8,−154.9]	[0.6700,0.6437,0.3699]
Measurement plane 3	57	[27.5,−205.8,−154]	[0.8412,0.5309,0.1031]
Measurement plane 4	59	[29.6,−204.5,−154]	[0.7632,0.6017,0.2357]
Input plane	63	[32.6847,−203.4475,−154.6588]	[−0.9440,−0.0722,0.3220]
Output plane 1	–	[0.0316,−0.2009,−0.1520]	[0.2656,0.0262,−0.9637]
Output plane 2	–	[0.0298,−0.2102,−0.1618]	[−0.0834,0.8633,0.4977]
Output plane 2	–	[0.0240,−0.2143,−0.1570]	[0.1206,0.6726,0.7301]
Output plane 2	–	[0.0196,−0.2107,−0.1569]	[0.9685,0.2220,0.1124]

Extracted cardiac cycle lengths vary for each cardiac cycle and each measurement. The patient is known to have an existing aneurysm in the MCA on the opposite (left) side, within which the velocity magnitudes could not be clearly resolved using TCD due to its unfavourable orientation. We segmented the images using VMTKlab (vmtklab.orobix.com), and voxelized the 3D model using the HemeLB setup tool. The resulting geometry has one inflow region and five outflow regions—two small ones at the top near the inflow boundary, two larger ones at the bottom, and the largest one left of the 49 mm plane (see Figure 1B).

The 2D inflow profiles were reconstructed from the 1D TCD data by mapping a parabolic profile to the non-circular inlets. This parabolic inlet profile has the original velocity from the 1D TCD data mapped to the centre of the inlet (the lattice site which is furthest from any wall), and 0 velocity values mapped to wall boundary sites. The velocity magnitude of a given lattice site is then calculated using a parabolic equation, which depends on the distance of the lattice site to the nearest vessel wall site in the inlet plane (0 for wall sites, 1 for the site in the centre, and values in between for other sites).

The boundary conditions in the lattice Boltzmann method were implemented as follows. To set the reconstructed velocity profile ${\vec{u}}_{\text{TCD}}\left({\vec{x}}_{\text{in}},t\right)$ at the inlet, we use a method introduced by Ladd (1994). A simple bounce-back boundary condition is augmented with a momentum term that results in a time-dependent Dirichlet condition for the velocity

(1) $$\vec{u}({\vec{x}}_{\text{in}},t)={\vec{u}}_{\text{TCD}}({\vec{x}}_{\text{in}},t).$$

**Figure 3:**
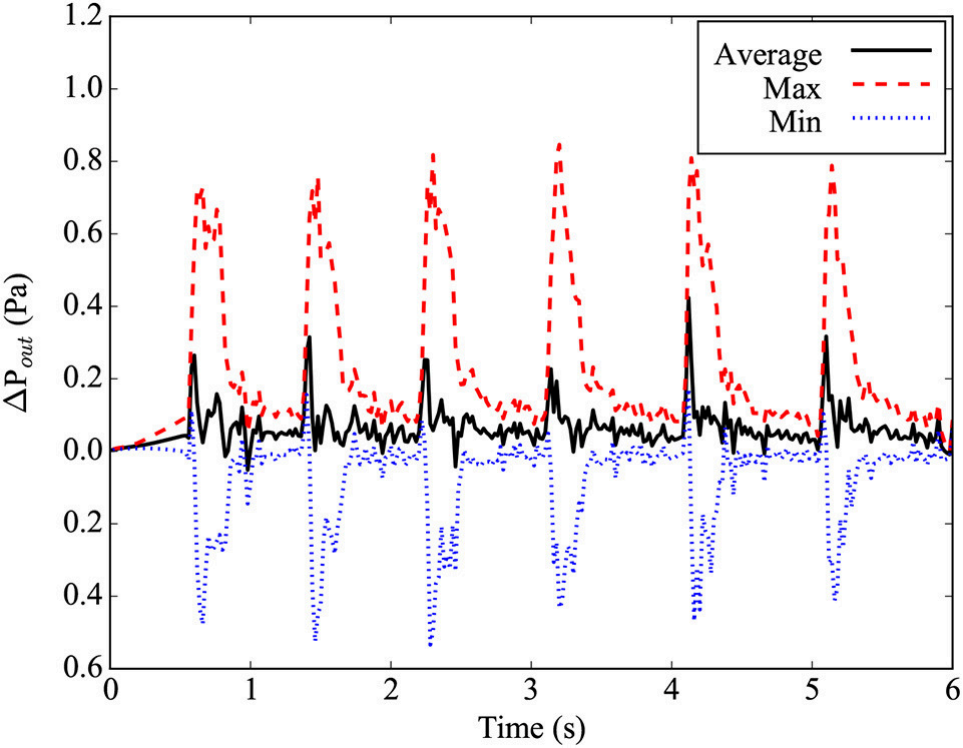
Differential pressure at the main outlet plane, relative to the ideal gas pressure for average density in the simulation. The maximal pressure found in the plane is given by the red dashed line, while the minimal pressure found in the plane is given by the blue dotted line. The average pressure in the plane is given by the black line.

At the outlet, we employed an open boundary condition in terms of a mixed Dirichlet-Neumann boundary condition (Nash et al., 2014)

(2) $${\vec{u}}_{p}({\vec{x}}_{\text{out}},t)=0,$$

(3) $$\hat{n}\cdot \nabla {\vec{u}}_{n}({\vec{x}}_{\text{out}},t)\;=0,$$

where $\hat{n}$ is the normal vector of the outlet plane, and ${\vec{u}}_{p}$ and ${\vec{u}}_{n}$ are the in-plane and normal components of the outlet velocity, respectively. The gradient in Equation (3) is taken as the first-order finite difference approximation on the lattice Boltzmann grid. In the implementation by Nash et al. (2014), the density $\rho \left({\vec{x}}_{\text{out}},t\right)$=ρ_0_ at the outlet is prescribed in order to determine the unknown fluid variables. It is worth noting that prescribing the density at the outlet fixes the static pressure through the ideal gas equation of state. However, this does not constrain the dynamic pressure which varies over a cardiac cycle as shown in Figure 3.

The shear-thinning behavior of blood is modeled using the Carreau-Yasuda (CY) model which employs the expression (Boyd et al., 2007; Bernabeu et al., 2013)

(4) $$\frac{\eta (\dot{\gamma })-\eta_{\infty }}{\eta_{0}-\eta_{\infty }}={\left(1+{\left(\lambda \dot{\gamma }\right)}^{a}\right)}^{\frac{n-1}{a}}$$

to account for the dependence of the dynamic viscosity η on the shear rate $\overset{{}^{\circ}}{\gamma }$. Here, η_0_ and η_∞_ are the asymptotic values at zero and infinite shear rate, and *a*, *n*, λ are empirical materials parameters that describe the shear-thinning curve. The CY model represents a smooth transition between Newtonian behavior at η_0_ and η_∞_.

The HemeLB simulations were performed on the ARCHER supercomputer at EPCC in Edinburgh, United Kingdom, and the SuperMUC supercomputer at LRZ in Garching, Germany. We used between 1,536 and 24,768 cores, depending on the chosen resolution.

### 2.1. Choice of lattice boltzmann parameters

Our lattice Boltzmann model uses a D3Q19 lattice with the Lattice Bhatnagar-Gross-Krook (LBGK) collision model (Bhatnagar et al., 1954). The relaxation parameters are set to yield the dynamic viscosity of blood of η = 0.004 Pa·s. The parameters used in the CY model are η_0_ = 0.16 Pa·s, η_∞_ = 0.0035 Pa·s, λ = 8.2 s, *a* = 0.64 and *n* = 0.2128 as given by Boyd et al. (2007) and previously adopted by Bernabeu et al. (2013). In our full-resolution, full-velocity simulations, we used a voxel size of 10 μm, a time step size of 0.28 μs, and a geometry consisting of 174,738,326 fluid sites. The simulations ran for 21.43 million time steps, which corresponds to 5 s of simulated time following a one-second “warmup” period (during which the inlet flow speed is increased gradually from rest in order to avoid flow instability or shockwaves resulting from a step change). The Reynolds number of our full-velocity simulation is approximately 966, based on a characteristic diameter of 24 mm with the highest measured peak velocity of 1.61 m/s.

We also performed simulations at reduced velocity and resolution, multiplying the velocities by 50 or 25%, as well as with increased voxel sizes of 20 and 40 μm. We discuss the implications of using this type of velocity scaling in detail in the next subsection.

### 2.2. Velocity scaling

The LBM is valid in the incompressible regime and introduces compressibility errors that scale quadratically in the Mach number *Ma* = *U*/*c*_*s*_, where *U* is the flow velocity and *c*_*s*_ the speed of sound. The cardiac flow is characterized by the Reynolds number *Re* = *UD*/ν and the Womersley number α = (ω^*D*^2^/ν)1/2^, where *D* is the vessel diameter, ν = η/ρ is the kinematic viscosity, and ω is the angular frequency of the oscillating flow due to the cardiac cycle. In terms of the simulation parameters, the kinematic viscosity of the lattice BGK model and the speed of sound are given by

(5) $$\nu \;=\frac{1}{3}\left(\hat{\tau }-\frac{1}{2}\right)\frac{{\left(\Delta x\right)}^{2}}{\Delta t},$$

(6) $$c_{s}\;=\frac{1}{\sqrt{3}}\frac{\Delta x}{\Delta t},$$

where $\hat{\tau }$ is the dimensionless relaxation parameter of the BGK model, and Δ*x* and Δ*t* are the discrete lattice spacing and time step, respectively. Based on the Reynolds and Mach numbers, we have the following relation for the dimensionless relaxation parameter

(7) $$\hat{\tau }-\frac{1}{2}=\sqrt{3}\frac{D}{\Delta x}\frac{Ma}{Re}.$$

Linear stability requires $\hat{\tau }>0.5$ which guarantees a positive viscosity. However, it is mandatory to keep the Mach number small in order to reduce compressibility errors and make the system less prone to instabilities due to density fluctuations. In the standard diffusive scaling, convergence is achieved by reducing the Mach number while keeping the Reynolds number constant. This implies (Δ*x*)^2^~Δ*t*. Thus, reducing the Mach number by means of increasing resolution results in an increase in computational costs due the cubic scaling of volume.

Therefore, some authors have been tempted to use lower flow velocities, e.g., from healthy subjects (Miura et al., 2013; Byrne et al., 2014), in order to maintain stable simulations at a larger voxel size Δ*x*. The ratio of the reduced velocity *U*′ and the original velocity *U* is denoted by a scaling factor *s*. This leads to a scaling relation

(8) $$s=\frac{U^{\prime }}{U}=\frac{\nu^{\prime }Re^{\prime }D}{\nu ReD^{\prime }}=\frac{\alpha^{2}\omega^{\prime }D^{\prime }Re^{\prime }}{{\alpha^{\prime }}^{2}\omega DRe},$$

where the prime denotes the quantities associated with the scaled velocity *U*′. If one insists on a fixed system size *D*′ = *D* and cardiac cycle length ω′ = ω, it is not possible to fix both the Womersley number and the Reynolds number at the same time such that the simulation is performed in a flow regime different to that of the full velocity simulation. In section 3.2, we demonstrate that this can significantly impact the simulated flow patterns.

## 3. Results and discussion

We present results from three types of simulation. First, we compare our full velocity and full resolution (10 micron voxel size) simulations against the TCD measurements on the same patient. Second, we present the results from simulations at reduced velocity and reduced resolution, and compare them both with results from our full-scale simulations and with the TCD measurements. Third, we compare the results of simulations using a Newtonian rheology model to simulations using a non-Newtonian (Carreau-Yasuda) rheology model (Abraham et al., 2005).

### 3.1. Validating full velocity haemodynamics predictions against measurements

**Figure 4:**
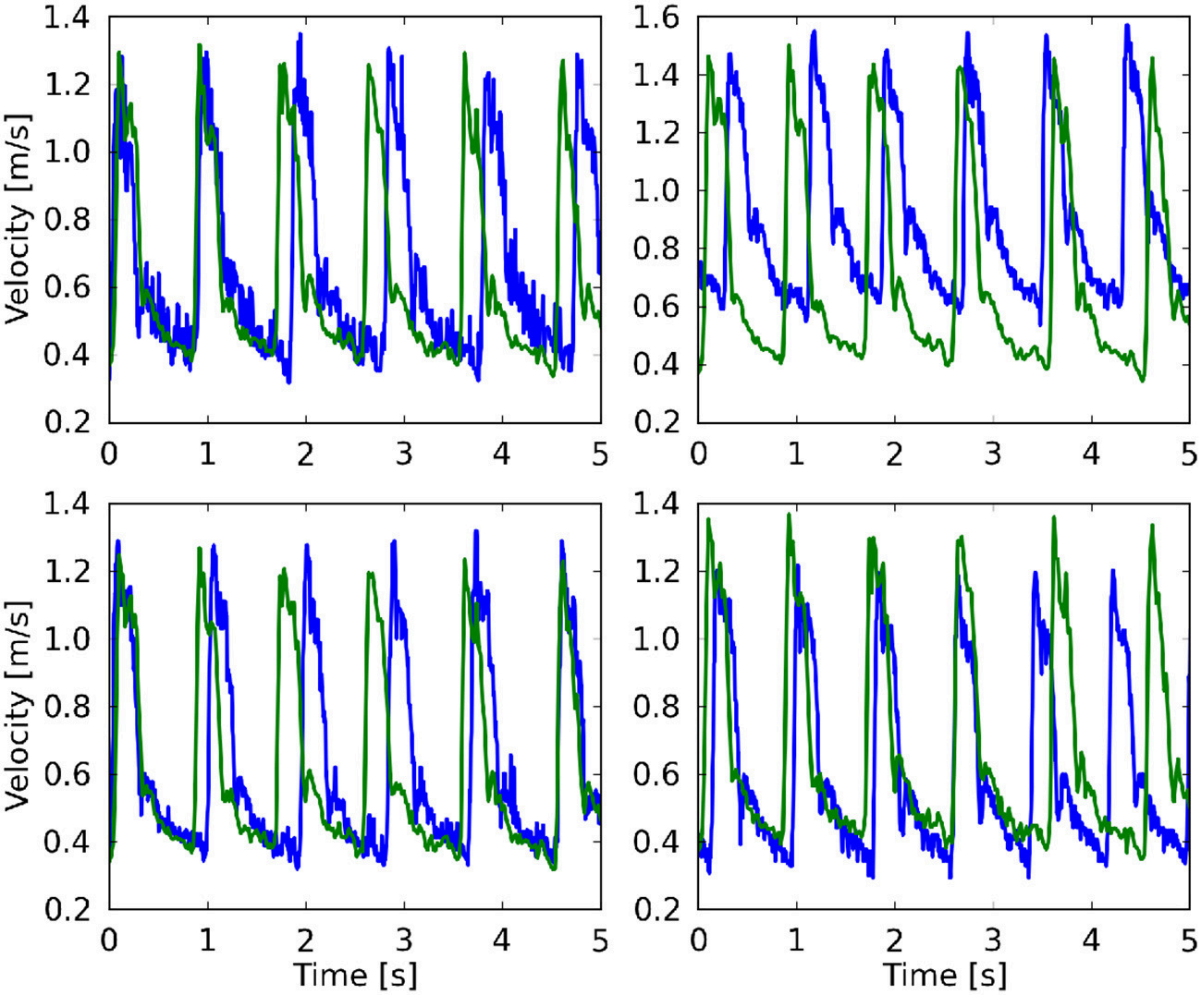
Comparison of the maximum velocity using both TCD (blue line) HemeLB (green line), given for the planes at 49 mm **(Top left)**, 54 mm **(Top right)**, 57 mm **(Bottom left)**, and 59 mm **(Bottom right)**. HemeLB results presented here are for the run at 100% velocity and with Newtonian rheology. The phase has been shifted to align both results with the start of the first cardiac cycle.

In Table 1 we present the maximum velocity *v*_max_ as measured with TCD and the simulation results for all four measurement planes. Our simulations predict the flow velocity with a relative error of less than 9% in all cases. In Figure 4 we present a direct comparison of our TCD measurements in the four planes over time, and our velocity predictions derived using HemeLB at the same locations. We observe good agreement between the simulation results and the measured TCD profile. The differences can be ascribed to the limitations of our approach (see section 3.4) and uncertainties in the measurements, including phase misalignments due to the sequential nature of the TCD measurement.

**Figure 5:**
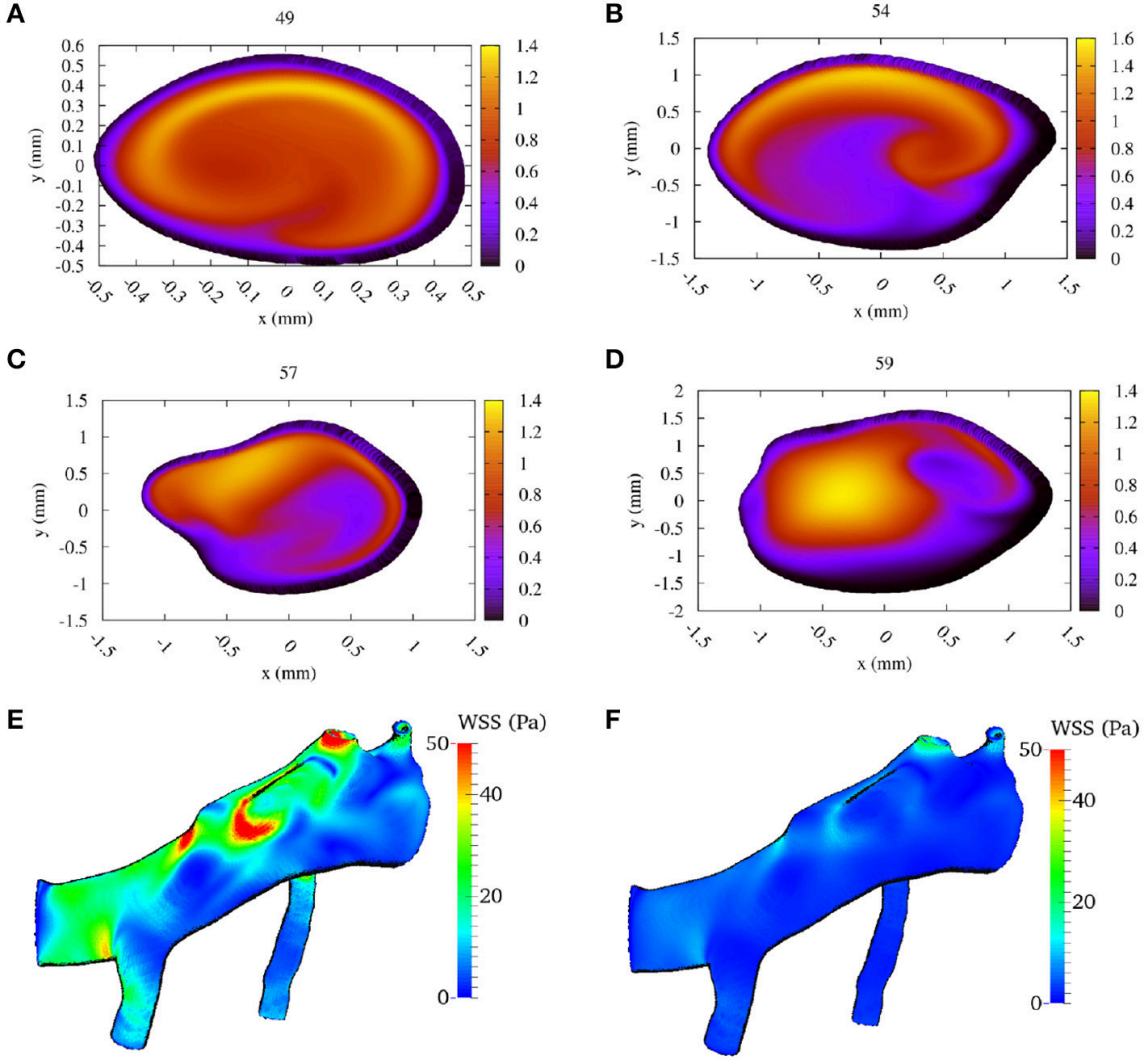
Calculated flow velocity magnitude, in the direction along the vessel center lines, at the second peak systole (at 1.44 s) in m/s for each of the four TCD validation planes. We show the velocity profiles in **(A–D)** for planes at a depth at 49, 54, 57, and 59 mm, respectively (run at 100% velocity, Newtonian rheology). We present the calculated wall shear stress (WSS) at peak systole in **(E)**, and at diastole (at 2.18 s) in **(F)** (using the same scale).

In Figures 5A–D, we present the two-dimensional velocity profiles extracted from the simulation at the four measurement planes. These profiles were extracted at the peak systole of the second cycle, corresponding to a velocity at the inlet of approximately 1.42 m/s. The figures show how the profile changes along the flow through the MCA. Compared to the inflow profile, the velocity profile at 59 mm is already substantially different, as a high velocity region is visible on the left side of the artery. The profiles at 57 and 54 mm show a strong concentration of (high) velocity near the top, which is presumably due to the bend present in that region of the artery, while a bend in the opposite direction just before the 49 mm plane is the likely cause of the more evenly distributed velocities there at peak systole (Figure 5E). In Figures 5E,F we show the calculated wall shear stress (WSS) across the MCA at peak systole and diastole (at 2.18 s). The front in Figures 5E,F corresponds to the left side in Figures 5A–D. We observe a WSS of >40 Pa during the systole in at least three locations. The WSS at the subsequent diastole (Figure 5F) remains relatively high at the location near the second outlet at the top, which indicates that this location could be susceptible to the formation of a new aneurysm.

### 3.2. Full vs. reduced velocity simulations

In this section we compare the velocity profiles at peak systole from simulations at 10 μm voxel size and full velocity with those at reduced velocity and/or increased voxel size. Reduced velocity and resolution runs are attractive because they are cheaper, faster to run, and more likely to become computationally tractable in a clinical setting. For example, at time of writing, a full velocity run across five cardiac cycles costs approximately £4200 on the ARCHER supercomputer (EPCC, 2017), whereas a run at 50% velocity and the same resolution costs £2100 and a run at 50% velocity and 20 μm voxel size costs £500 to perform. However, reduced velocity simulations have a lower Reynolds number which affects a wide range of flow properties. In this study we have performed runs at 50% velocity (Re ~ 483) and 25% velocity (Re ~ 242).

**Figure 6:**
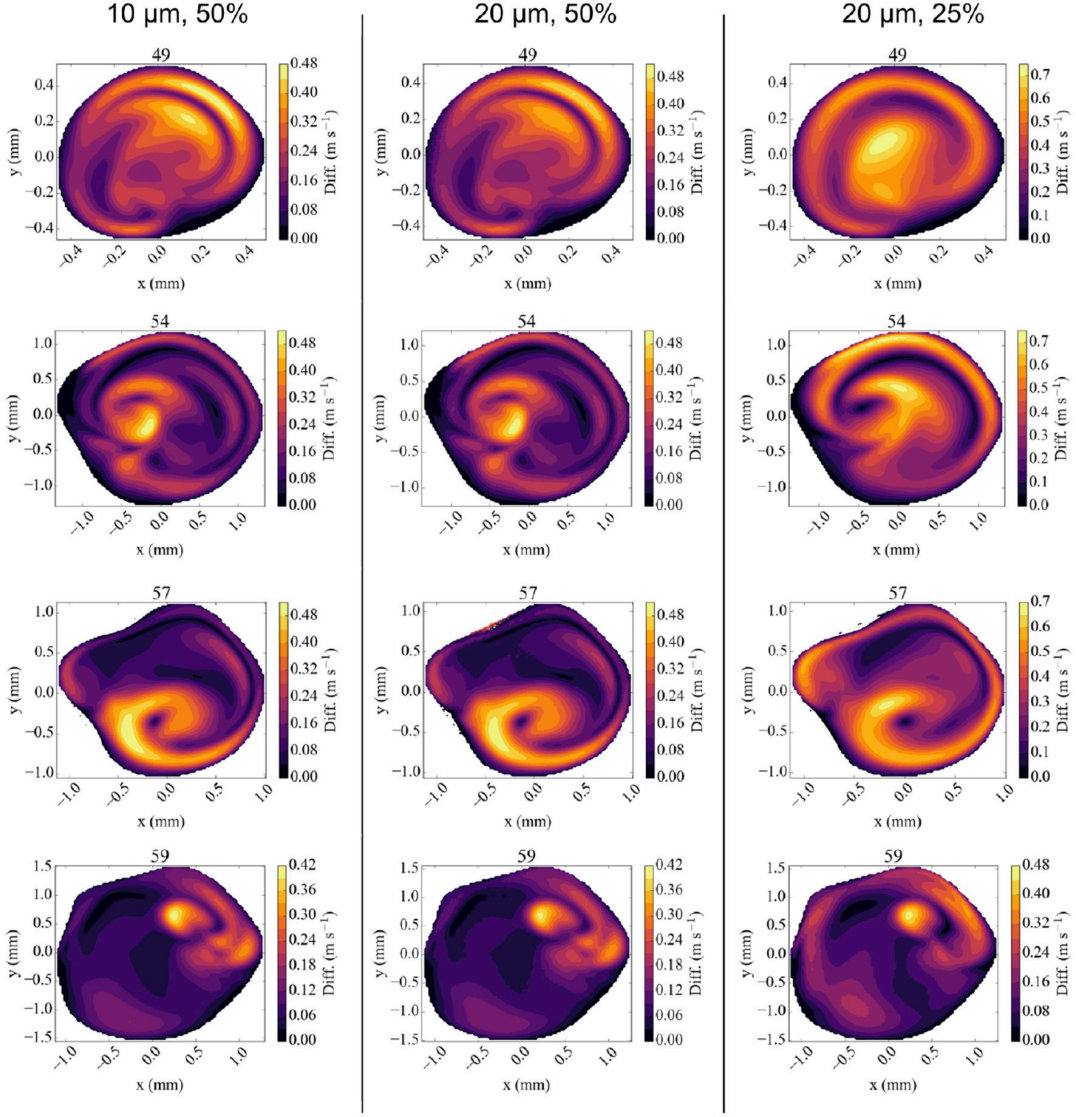
Absolute difference in flow velocity, between the run with Newtonian rheology at 10 μm resolution and 100% velocity and other runs for each of the four validation planes. Comparisons are made with runs at 10 μm and 50% velocity **(Left column)**, 20 μm and 50% velocity **(Middle)**, and 20 μm, and 25% velocity **(Right)** respectively. The velocities in reduced velocity runs are multiplied by 2 (for the 50% velocity runs) or 4 (for the 25% velocity runs). The snapshots were made at the second peak systole (at 1.44 s), with differences in m/s.

We compare our simulation results at full velocity and resolution with those at reduced velocity and resolution in Figure 6 and Table 3. When we reduce the inflow velocity by 50%, the maximum inflow velocity at the inlet is 0.75 m/s (not an uncommon value for healthy volunteers) (Bishop et al., 1986) instead of 1.50 m/s (not an uncommon value for stroke patients) (Manno et al., 1998). We multiply the extracted velocities from our reduced velocity runs by two for simulations at 50% inflow velocity, and by four for simulations at 25% velocity. When comparing the runs with full inflow velocity runs with those at 50%, we already observe major differences in the extracted velocities. Here the comparisons at all four locations feature velocity differences of more than 0.4 m/s, and more than 30% of the maximum absolute flow velocity extracted in the corresponding plane. For the planes at 49 and 57 mm we see very large velocity differences near the vessel wall. This is likely due to the much higher Reynolds number of the flow in the full velocity run. When we compare the rescaled 50% velocity runs to the TCD measurements, the velocities differ by up to 15.5%, which is almost twice as large as the 8.8% maximum difference between TCD measurements and full velocity runs.

**Table 3:** Comparison of full and reduced velocity simulations against TCD velocity measurements.

**v scaling**	**Rheology**	**Voxel size**	$v_{\text{max}}^{sim}$ **at depth [mm]**				***d***_*****r*****_ **[%] at depth [mm]**			
**(%)**		**[μm]**	**49**	**54**	**57**	**59**	**49**	**54**	**57**	**59**
100	Newton	10	1.32	1.50	1.27	1.37	−7.7	−6.8	−3.8	+8.7
100	CY	10	1.32	1.51	1.27	1.37	−7.7	−6.2	−3.8	+8.7
50	Newton	10	1.26	1.36	1.22	1.34	−11.9	−15.5	−7.6	+6.3
50	Newton	20	1.25	1.36	1.22	1.34	−12.6	−15.5	−7.6	+6.3
50	CY	20	1.25	1.36	1.22	1.34	−12.6	−15.5	−7.6	+6.3
25	Newton	20	1.26	1.22	1.15	1.27	−11.9	−24.2	−12.9	+0.8
TCD measurement			1.43	1.61	1.32	1.26	−	−	−	−

*We present the velocity scaling used in each run in column 1 (100% for a full velocity run), the rheology model used in column 2, the voxel size used in column 3 (10 μm for a full resolution run), the extracted peak velocity in each of the measurement planes in columns 4 to 7, and the relative difference in peak velocity compared to TCD measurements for each plane in columns 8 to 11. As a reference, we provide $v_{\text{max}}^{\text{TCD}}$ for each of the planes in the bottom row*.

The results of the 50% velocity run with 20 μm voxel size are almost identical to the one with 10 μm voxel size, with only very small differences in all the velocity planes. However, the run with 25% velocity is considerably less accurate, with absolute velocity differences up to 0.75 m/s, in particular close to the vessel walls. These errors are still smaller close to the inflow boundary at 59 mm, but dominate the overall result in the validation planes that are beyond the bifurcation with lenticulostriate arteries.

We conclude that simulations with reduced velocities affect the accuracy of the results significantly. This is particularly important because realistic velocities close to the wall are essential to obtain accurate wall shear stress estimates. We find that no such estimates can be reliably made for half velocity runs.

### 3.3. Comparing rheology models

To compare different rheology models, we performed simulations on our MCA geometry using a Carreau-Yasuda (CY) rheology model (Abraham et al., 2005). When the CY model was adopted, Bernabeu et al. found important differences in the WSS and Three-Band-Diagram analysis outcome for the MCA under “healthy human” flow conditions. Here we focus on differences in velocities obtained from the two rheology models, as we are interested in comparing simulation predictions to TCD measurements.

**Figure 7:**
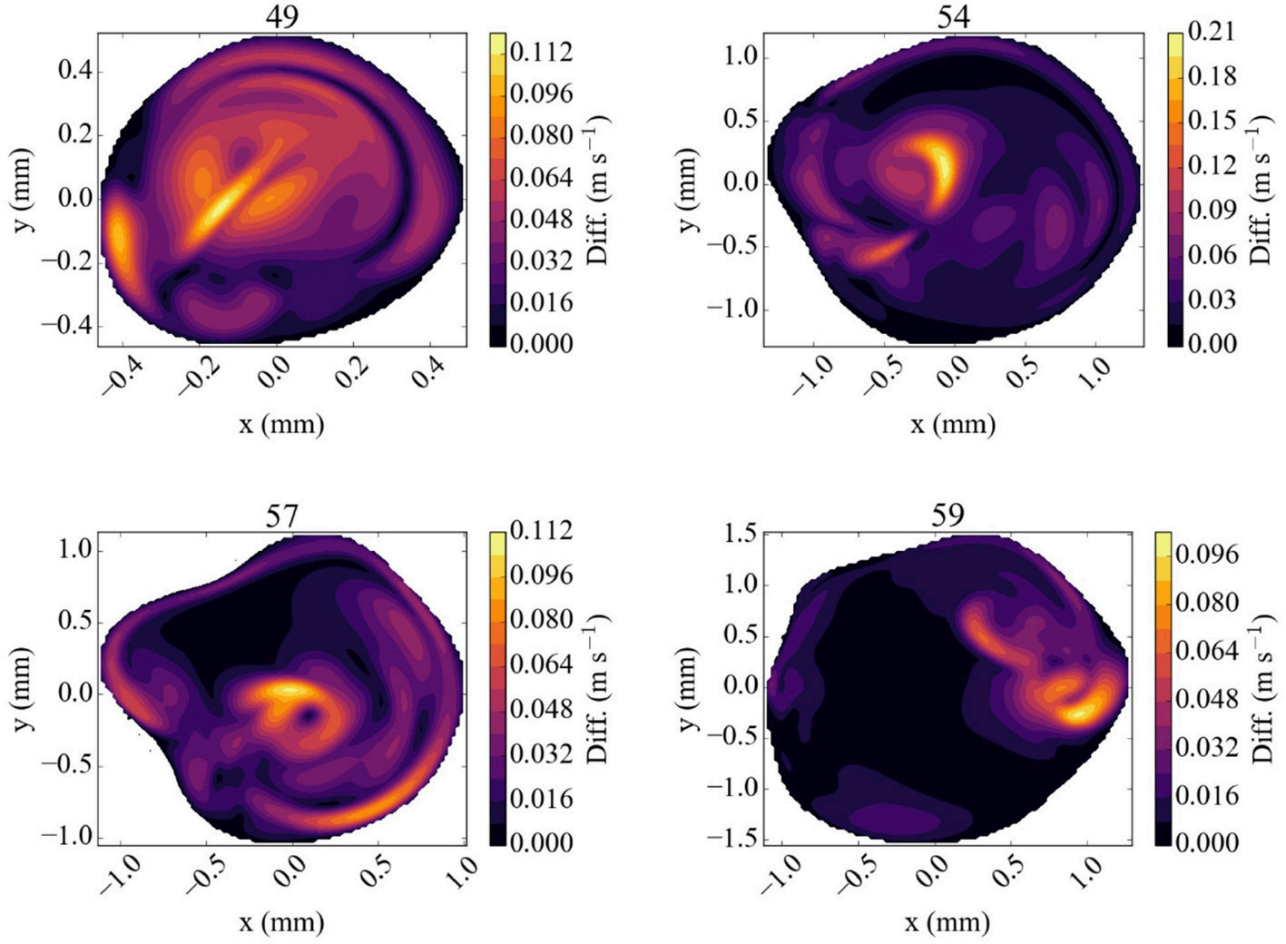
Absolute difference in flow velocity, between the run with Newtonian rheology and run with CY rheology. Both of these runs were performed at 100% of the full velocity. The snapshots were made at the second peak systole (at 1.44 s), with differences in m/s, for each of the four TCD validation planes.

The difference in flow velocity between the Newtonian rheology model and the CY rheology model at peak systole is shown in Figure 7. We observe differences in velocity of up to 0.12 m/s in three of the four validation planes, and a difference of up to 0.21 m/s in a highly concentrated central region in the 54 mm measurement plane. In all cases the velocity differences are largest in regions where the absolute velocity is relatively small in the Newtonian rheology results, cf. Figure 5, while only smaller differences exist in regions where the velocity is relatively large. These results suggest that the choice of using either a CY or Newtonian rheology model has little effect on $v_{\text{max}}^{\text{sim}}$ in all our comparisons (see Table 3).

**Figure 8:**
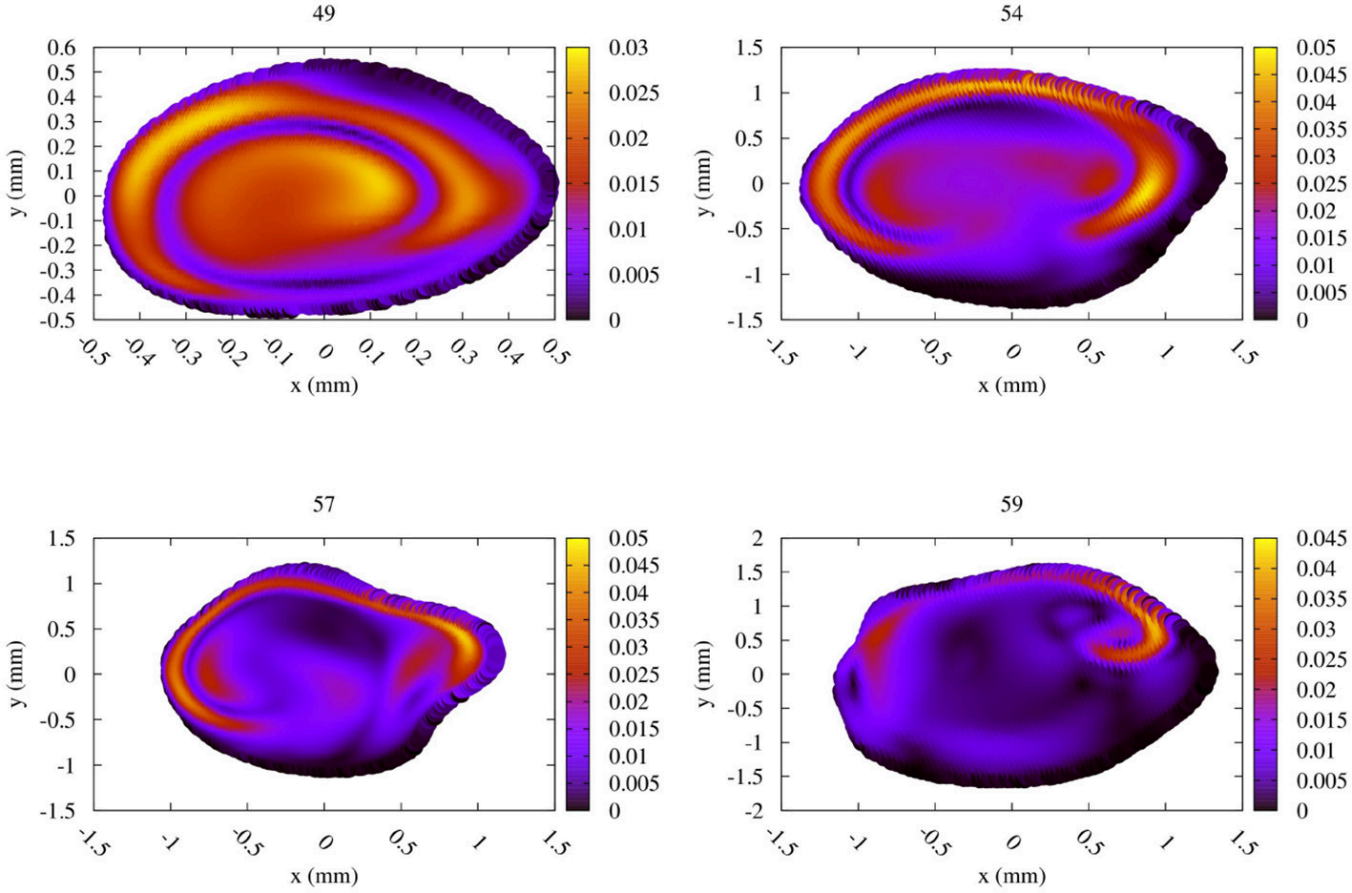
Absolute difference in flow velocity, between the run with Newtonian rheology and run with CY rheology. Both of these runs were performed at 50% of the full velocity, with the differences rescaled by a factor 2. The snapshots were made at the second peak systole (at 1.44 s), with differences in m/s, for each of the four TCD validation planes.

The difference between the Newtonian and the CY rheology model for 50% reduced velocity is shown in Figure 8 at peak systole. As noted above, velocity extractions from runs at 50% velocity are multiplied by 2 to enable a direct comparison with full velocity runs. The difference in velocities between the 50% runs is considerably smaller than for 100% velocity runs, reaching at most 0.05 m/s in any of the measurement planes. The velocity difference is largest close to the arterial wall, but is in all cases much smaller than the velocity mismatch introduced by the velocity reduction (see Figure 6, left row). This is in line with the finding that the choice of the rheology model has a small effect, and in the reduced velocity runs the impact of scaling down the velocity on accuracy is the dominating factor.

### 3.4. Limitations of our study

The main limitations of our validation study are related to data acquisition, model construction and simulation constraints.

Regarding TCD measurements, phase misalignments are common when directly comparing simulation results to these measurements, due to differences in apparent cardiac cycle length between the consecutively measured TCD planes (see Figure 4). Furthermore, due to the proprietary nature of the TCD numerical data, numerical velocity values were extracted semi-automatically from JPEG images obtained with the Doppler BoxX software, which may introduce small transcription errors of up to 0.0064 m/s due to the resolution of the images. The measurement quality and level of background noise can vary with different measurements, as different depths are subject to varying levels of occlusion and wave propagation interference.

In the area of segmentation it is particularly challenging to accurately reproduce the small lenticulostriate arteries originating near the origin of the MCA (Kang et al., 2012). These arteries are not always clearly captured in the medical imaging data, and many existing haemodynamics models of MCAs do not include them, while our geometry contains two of these arteries. However, omitting them altogether can lead to velocity overestimations in the remainder of the MCA. In our model we were able to resolve the lenticulostriate arteries to a limited extent after extensive segmentation efforts.

Due to the one-dimensional nature of the TCD measurement, we used a parabolic inflow velocity profile and fitted it to the non-circular shapes of the inflow boundaries (see section 2). Real inflow velocity profiles can vary depending on the morphology of the arterial network, as shown for example by Takeuchi and Karino (2010). Regarding the outlets, a more physiologically accurate choice of boundary condition would take into account the downstream peripheral resistance. However, such an approach introduces additional patient-specific parameters. For the purposes of the validation conducted in this study we intentionally limit the complexity of the model and thus use a simple mixed Neumann-Dirichlet boundary condition.

Furthermore, our simulation model is based on a rigid geometry and does not include elastic deformations of the vessel. In the case of blood flow in cerebral aneurysms, Dempere-Marco et al. (2006) found that incorporating wall motion has relatively little effect on the WSS. Understanding the dynamical response of arterial walls in the MCA, on a patient-specific level, is a particularly challenging area of research. However, recent studies show promising results that should soon allow us to examine these processes (Oubel et al., 2010; Vanrossomme et al., 2015).

### 3.5. Future work

There are a range of factors that we seek to incorporate in our model as part of our future research. Firstly, we aim to develop techniques to create more realistic inflow profiles by using simulation data of arteries upstream from the patients MCA. Secondly, we seek to enhance our model by incorporating mechanisms for arterial wall deformations and damage. Such mechanisms are highly complex and very difficult to measure experimentally, and therefore modelling them is a particularly challenging area of research. Thirdly, we seek to provide more realistic outflow properties by extending our geometry to arteries further downstream. This could be accomplished for example by investigating how existing (1D) resistance models could be accurately applied within the context of complex 3D simulation models, or by attempting to simulate the full human brain in 3D over realistic time scales, and using patient-specific flow conditions.

## 4. Conclusions

We have conducted a validation study comparing flow velocities from patient-specific lattice-Boltzmann simulations to clinical TCD measurements in the MCA. As part of the study, we analyzed simulation results obtained at reduced velocities and variable resolution. Moreover, we investigated the impact of using the Carrueau-Yasuda rheology model compared to a Newtonian rheology model.

We achieved very good agreement of the maximum velocity between our full patient-specific velocity simulation results and TCD measurements, with an error of less than 9% independent of the choice of rheology model. Simulating blood flow at reduced velocities, for example by scaling down the velocity or using velocity measurements from healthy subjects, is attractive because the simulation runs are computationally cheaper and deliver results faster. However, we found that scaling down the flow velocities leads to substantially larger errors, and an accurate comparison between simulations and TCD measurements is no longer achieved. Adopting a CY rheology model instead of a Newtonian one results in small changes in maximum velocities in the planes and in our validation, whereas substantial flow velocity differences are observed near the arterial wall and in the resulting WSS. However, the CY rheology model does not enable a significant improvement when the velocity is already scaled down (e.g., by using inflow profiles of healthy volunteers or reduced velocity Womersley profiles), as errors caused by this velocity scaling then dominate the overall accuracy. Figures 7, 8 suggest that a Newtonian rheology model may be a justifiable approximation for MCA simulations at lower (i.e., < 0.75 m/s) peak flow, but that this could quickly become problematic for the higher flows typically recorded in unhealthy patients (in which 1.5 m/s is not unusual).

Computational haemodynamics predictions that accurately match patient-specific TCD measurements are likely to become an important asset in clinical settings and pave the way to using computer models in the process of clinical decision making (Fenner et al., 2008; Sadiq et al., 2008). Compared to clinical measurements alone, patient-specific simulations allow us to obtain information about a much wider range of flow properties, such as detailed flow velocity characteristics and wall shear stress estimates. In addition, simulations can help predict flow velocity in areas that have not been directly measured, and thereby help reduce the number and intensity of invasive measurements that need to be performed. Here we have shown that a combination of non-invasive TCD measurements with haemodynamics simulations can lead to accurate predictions of blood flow velocity throughout the MCA. The ability to make these accurate predictions constitutes an important step in making computational haemodynamics a viable approach for assessing intracranial blood flow.

## Ethics statement

The patient-specific data (3D angiography, TCD measurements) used in this study was available on the shelf and did not contain identifiable information. The segmented geometry is already published Itani et al. (2015). The present study involved only secondary analysis of de-identified data that is not linked to the subjects from whom it was originally collected.

## Author contributions

DG conceived the study, while DG and RR carried out the simulations, performed the validation comparison, and wrote the manuscript. US advised on the choice of simulation parameters and contributed to writing the manuscript. RC segmented the medical images and extracted the TCD velocity profiles from the measurement images, with help from DG, US, and PC. FR obtained the original angiography images, while HC performed the TCD measurements. Both FR and HC advised on the medical aspects of the manuscript. PC coordinated the study and helped draft the manuscript. All authors gave final approval for publication.

### Conflict of interest statement

The authors declare that the research was conducted in the absence of any commercial or financial relationships that could be construed as a potential conflict of interest.

## Supplemental data

We provide the source data for our publication via Figshare under doi: 10.17633/rd.brunel.5001962.
